# Introductory and Other Addresses

**Published:** 1853-05

**Authors:** 


*Introductory and other Addresses.* — So large a number of publications falling under the above class have accumulated on our table, that we shall be obliged to content ourselves with but little more than their enumeration with their respective titles, and a general acknowledgment of the courtesy of their authors in favoring us with copies.

1. *A Discourse on the Times, Character and Writings of Hippocrates.* By Elisha Bartlett, M. D., Prof. of Materia Medica and Therapeutics in the College of Physicians and Surgeons, N.Y. Published by the class.

This is an elaborate discourse, and as a rhetorical performance highly finished.

2. *Inaugural Address of Worthington Hooker, M. D., as Professor of the Theory and Practice of Medicine in Yale College.*

The subject of this address is "The present mental attitude and tendencies of the medical profession."

3. *A Lecture on the Treatment of Disease.* By Jacob Bigelow, M. D., Prof. of Materia Medica and Clinical Medicine in Harvard University.

A sound, sensible discourse, which every practitioner may read with advantage.

4. *Certainty in Medicine.* By James Bryan, M. D., Prof. of Surgery.

A well written address.

5. *Sketches of Military Surgery: an Introductory Discourse delivered to the Kentucky School of Medicine.* By Prof. J. B. Flint.

In this discourse Prof. Flint proposes a new method of making medical appointments for the army and navy. The method consists in the delegation of power to the faculty of every medical school of good standing, in the United States, annually to confer upon one, two, three or more, as need may be, of the most meritorious and accomplished of their graduates, certificates of qualification for army and navy preferment, the delivery of these certificates to make a part of the public exercises of the commencement. He urges the merits of this plan with cogent arguments.

6. *The incentives, means and rewards of Study: an Introductory Address, delivered at the opening of the 23d Annual Course of Lectures in the Med. College of Ohio.* By L. M. Lawson, M. D.

A learned and philosophical discourse.

7. *An Introductory Lecture read before the class in Starling Medical College.* By Robert H. Paddock, M. D., Prof. of Anatomy and Physiology.

8. *Professor Bache's Introductory Lecture, delivered in Jefferson Medical College of Philadelphia.*

9. *Professor Bache's Valedictory Address to the Graduates of Jefferson Medical College.*

10. *Lecture Introductory to the Second Course in the Medical Department of the University of Nashville.* By W. K. Bowling, M. D., Prof. of the Institutes and Practice of Medicine.

In this discourse the author, very naturally, discusses the prospects of the institution recently established at Nashville, and augurs future prosperity from the success of the two first sessions. Believing that honorable competition is not of disadvantage in medical education, more than in other departments of human effort, we say God speed! The field is open and broad enough. Ferat qui meruit palmam.

11. *Biographical Sketch of J. Kearny Rogers, M. D.* By Edward Delafield, M. D.

In this excellent biographical sketch we find a passage which provokes criticism:

Prior to describing the operation for tying the subclavian as performed by Dr. Rogers, the author says: "He did at last arrive at one great object of every surgeon's ambition, the successful performance of a great and difficult operation which had been attempted before by the greatest masters of his art, but without success." An account of the tying the subclavian follows, which the writer characterizes as remarkable and successful, albeit the patient died the 15th day after the operation, of arterial hæmorrhage! We claim that the sentiment conveyed in the foregoing quotation is a libel on the true surgeon, and we are at a loss to perceive how the shortening of a poor patient's life by tying the subclavian is to be viewed as the "crowning act of the surgical life" of the operator.

12. *History of the Medical Department of the University of Louisville; an Introductory Lecture.* By LUNSFORD P. YANDELL, M. D., Professor of Physiology and Pathological Anatomy.

As the title purports, this is a historical sketch of the University of Louisville by one connected with the institution from the beginning, and to whose efforts it is much indebted for its past and present prosperity. The address is written in the easy, graceful style for which the author is distinguished.

13. *Introductory Address delivered by Professor J. C. Hughes, before the class of medical students at the College of Physicians and Surgeons of the Iowa University.*

This address contains many interesting facts culled from the history of medical science.

